# Predictors of Clinical Inertia and Type 2 Diabetes: Assessment of Primary Care Physicians and Their Patients

**DOI:** 10.3390/ijerph19084436

**Published:** 2022-04-07

**Authors:** Nemanja Isajev, Vesna Bjegovic-Mikanovic, Zoran Bukumiric, David Vrhovac, Nebojsa M. Lalic

**Affiliations:** 1 Centre School of Public Health and Management, Faculty of Medicine, University of Belgrade, dr Subotica 15, 11000 Belgrade, Serbia; vesna.bjegovic-mikanovic@med.bg.ac.rs; 2 Institute of Medical Statistics and Informatics, Faculty of Medicine, University of Belgrade, dr Subotica 15, 11000 Belgrade, Serbia; zoran.bukumiric@med.bg.ac.rs; 3 University Children’s Hospital, Tirsova 10, 11000 Belgrade, Serbia; david.vrhovac@gmail.com; 4 Clinic for Endocrinology, Diabetes and Metabolic Diseases, Faculty of Medicine, University of Belgrade, dr Subotica 13, 11000 Belgrade, Serbia; nebojsa.lalic@med.bg.ac.rs

**Keywords:** diabetes type 2, clinical inertia, diabetes care, physician inertia, primary care physicians, glycemic control

## Abstract

With the growing prevalence and complex pathophysiology of type 2 diabetes, many patients fail to achieve treatment goals despite guidelines and possibilities for treatment individualization. One of the identified root causes of this failure is clinical inertia. We explored this phenomenon, its possible predictors, and groups of patients affected the most, together with offering potential paths for intervention. Our research was a cross-sectional study conducted during 2021 involving 52 physicians and 543 patients of primary healthcare institutions in Belgrade, Serbia. The research instruments were questionnaires based on similar studies, used to collect information related to the factors that contribute to developing clinical inertia originating in both physicians and patients. In 224 patients (41.3%), clinical inertia was identified in patients with poor overall health condition, long diabetes duration, and comorbidities. Studying the changes made to the treatment, most patients (53%) had their treatment adjustment more than a year ago, with 19.3% of patients changing over the previous six months. Moreover, we found significant inertia in the treatment of patients using modern insulin analogues. Referral to secondary healthcare institutions reduced the emergence of inertia. This assessment of primary care physicians and their patients pointed to the high presence of clinical inertia, with an overall health condition, comorbidities, diabetes duration, current treatment, last treatment change, glycosylated hemoglobin and fasting glucose measuring frequency, BMI, patient referral, diet adjustment, and physician education being significant predictors.

## 1. Introduction

The global burden of type 2 diabetes is steadily increasing with a 1.86 annual percent change of Disability Adjusted Life Years (DALYs) [1] and expectation to reach 783 million people living with diabetes in 2045 [2]. Similarly, data from Serbia additionally support these trends of a dramatic increase in the number of people living with type 2 diabetes. According to the latest data available from 2020, published by the Institute of Public Health of Serbia, the estimated number of patients was about 770 thousand, with a prevalence of 9%. In other words, 12% of the adult population is affected by diabetes. Even more important is that 43% have not been diagnosed. As expected, the prevalence was growing with age, and it is estimated that almost half of the patients are over 65 years of age [3]. The total DALYs attributed to this disease is 4.81% (1.94 annual percent change of DALYs) [1]. The growing prevalence of type 2 diabetes leads to increasing costs of prevention, diagnostics, and therapeutical activities [2].

With such a burden of type 2 diabetes, many studies explore complex pathophysiology, guidelines, and possibilities for treatment improvement. The cornerstone of the modern approach to treating type 2 diabetes is presented by UKPDS (UK Prospective Diabetes Study), which showed that tighter glycemic control is associated with a better treatment outcome and fewer complications [4]. Recognized world guidelines by the American Association of Diabetes (ADA) and European Association for the Study of Diabetes (EASD) agree that target values of glycosylated hemoglobin (HbA1c) that we should strive for in treatment in most patients should be ≤7% [5,6]. The common feature of all these recommendations is a focus on a faster treatment change if patients deviate from the targeted values. These corrections imply more frequent changes but greater possibilities of combining different antihyperglycemic agents than was previously possible.

Additionally, the ADA guide considers the existing cardiovascular disease, and thus choosing the appropriate treatment drug that can potentially reduce the risk of further disease progression [5]. In Serbia, national recommendations for the treatment of diabetes also exist, collected in the National Guideline from 2012. This guide emphasizes the importance of establishing metabolic control, expressed through the values of glycosylated hemoglobin (HbA1c) ≤ 7%, with an individual approach to the patient and the necessary rapid correction of the treatment to establish good disease control as soon as possible [7]. Provided health care and the healthcare system are defined by the Serbian Law on Health Care and conducted as per the National Guideline. A general practitioner is the first line for examining patients and establishing the diagnosis. A general practitioner can initiate oral antidiabetic medication and refer patients to the specialist in the primary healthcare center or the secondary-level institution. The general practitioner performs follow-ups, therapy modifications, and adjustments for oral antidiabetic drugs (OAD) treatment, and for other initiating and modification of injectable therapy, such as insulins, patients are referred to the specialist. The costs of therapy including metformin, sulphonylurea derivates, and insulins are covered by the National Healthcare Insurance Fund, while GLP-1 Ras, SGLT-2 inhibitors, and DPP-4 inhibitors are financed out-of-pocket and can be purchased based on physicians’ prescriptions [8,9].

Regretfully, in regular clinical practice, despite individualizing treatment and clear recommendations, it is very difficult to achieve the set goals from the guidelines. There are many reasons for this situation, and one of them is covered by the term “clinical inertia.” The term clinical inertia first appeared in the work of Phillips et al. in 2001. The authors first defined the term as the “absence of a new drug administration or a change in the dose of an existing drug when necessary” [10]. The work of Reach et al. from 2017 went a step further by linking the conditions, considered prerequisites to competencies, demonstrated by talking about clinical inertia with the individual patient and to meet his needs [11]. Therefore, a more comprehensive definition has been proposed, according to which clinical inertia is a case where a guideline is available, and the physician is aware of it, but he/she does not comply with its recommendations, although there are possibilities to apply them [11].

In treating type 2 diabetes, the phenomenon of clinical inertia is extremely important. The UKPDS study emphasized that achieving good metabolic control by appropriate treatment will consequentially lead to reducing the complications of diabetes. A decrease in HbA1c led to a 37% reduction in microvascular complications and a 21% reduction in the risk of death [4]. However, some physicians fail to follow recommendations for treatment improvement.

Looking at the challenges of clinical inertia, numerous studies indicated that correction of treatment, omitted when necessary, leads to poor glycemic control [12,13,14]. The work of Marrett et al., which included primary healthcare physicians, showed that one-third of elderly patients with type 2 diabetes who do not receive pharmacological treatment six months after diagnosis have poor disease control, with glycosylated hemoglobin values >8% [12]. The large multinational SOLVE study, which included 17,000 patients whose average glycosylated hemoglobin value at baseline was 8.9%, despite the treatment with a combination of oral antidiabetic drugs [13], has resulted in similar conclusions. In this light, the work published by Khunti et al., a retrospective study of a cohort of over 81,000 people with type 2 diabetes, showed that, in patients treated with one, two, or three oral antidiabetic drugs, time to the intensification of treatment with an additional oral agent or insulin was 7.2 years, pointing to significant clinical inertia [14].

Factors that can lead to clinical inertia can be categorized into three groups [15]. The first group includes factors that depend on the physician: the absence of clear treatment goals, the failure to initiate the treatment, the failure of titration, and others. The second group is factors that depend on the patient, such as low-health literacy or lack of trust in physicians. The third group refers to the health system and includes factors related to the existence of the register, the existence of guides, and the appropriate organization of health service.

The scope and degree of the clinical inertia prevalence in everyday practice in Serbia was unknown. Therefore, this study aims to explore the phenomenon of clinical inertia, determine its possible predictors and the groups of patients affected the most, and offer potential paths for intervention.

## 2. Materials and Methods

### 2.1. Study Design and Ethical Approval

The research design was a cross-sectional study conducted on the territory of the City of Belgrade in primary healthcare (PHC) institutions. The study participants were physicians and patients, while the data source was questionnaires based on similar studies of clinical inertia [15,16] and patients’ health records. The start of the data collection was initially planned for March 2020, but, due to the COVID-19 pandemic, had to be postponed, so active research was conducted from September 2020 to July 2021 in the premises of PHC centers in Belgrade, with one interruption during winter 2020.

Research started upon obtaining ethical approvals by PHC ethical boards and the Ethical Board of the University of Belgrade, Faculty of Medicine (decision No: 1550/IX-10, date: 25 September 2019).

### 2.2. Population, Sample Size and Procedures

**Figure 1:**
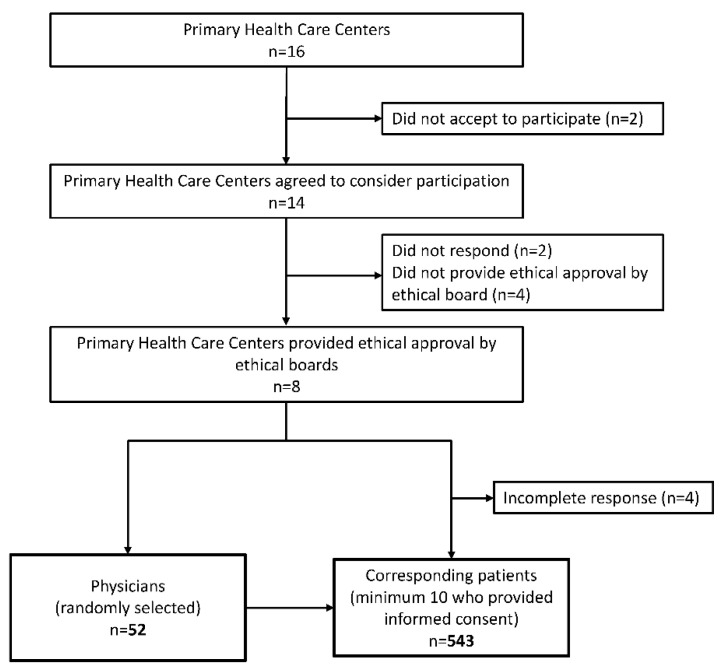
Participants’ flow-chart—recruitment of physicians and their patients.

Out of all 16 PHC centers considered for the research, two centers declined the request for participation due to the burden of regular work (Figure 1). The remaining 14 PHC centers that received the request for participation and agreed to consider it had to submit ethical approval for the study from their ethical boards. As 6 PHC centers did not submit ethical approval, research took place in 8 PHC centers.

The sample size of the investigated physicians was determined based on the assumed clinical inertia from other studies [14,15,16,17,18]. A minimum number of respondents, with an accuracy of 0.15, a confidence coefficient of 0.95, and an assumed frequency of the investigated phenomenon (22.1% to 55.7%), was 42 respondents for Belgrade. To achieve the required number of physicians in PHC centers where ethical approval has been received, participants—physicians were selected randomly; in total, 52 involved physicians. Each involved physician collected data from a minimum of 10 of their patients with type 2 diabetes (the first ten who fulfilled the inclusion criteria and agreed to participate in the study by signing an informed consent agreement). Inclusion criteria for patient participation were age ≥ 18 years and diagnosis of type 2 diabetes. Respondents fulfilling the inclusion criteria entered the study after introduction to the survey, research objectives, and signing informed consent. In this way, the patient sample involved 543 participants (Figure 1).

### 2.3. Research Instruments

The research tools used in this study were the physician’s questionnaire based on similar studies [15,16] and the patient’s questionnaire, designed according to studies that investigated the use of diabetic healthcare, adherence to treatment, and patient satisfaction [17,18].

The physician’s questionnaire collected data that indicate the emergence of clinical inertia and three groups of factors (patients’ characteristics, disease characteristics, and characteristics of the diabetic service organization), potentially leading to clinical inertia [17,18]. This questionnaire consisted of the basic information, such as socio-demographic data (age, marital status, standard of living, income), anthropometric data (height, weight, waist circumference), lifestyle (physical activities, diet, smoking), increased body weight, and obesity and health self-assessment, data related to the patient (duration of type 2 diabetes, laboratory analyses for the previous and current visit—the value of morning glycemia, lipids, glycosylated hemoglobin—HbA1c, measurements of glucoregulatory parameters, current treatment, last change of treatment, the occurrence of hypoglycemia, presence of comorbidities, and complications of type 2 diabetes). The questionnaire also contained data on the organization of diabetic care—the availability of diabetes counseling service and laboratory in PHC center, as well as other treatment information including average duration of the examination, potentially established treatment goals agreed upon with the patient, patients—referrals for further treatment (primary or tertiary level), information about patient education, defined patient visit plans, and patient records (existence of a special file of people with diabetes).

The patients’ questionnaire contained information about the factors contributing to the development of clinical inertia that originated from patients. Besides socio-demographic characteristics and lifestyle (diet and physical activity), the first section related to the disease, including current treatment, hypoglycemia, and comorbidities (changes in the fundus, high blood pressure, diabetic foot, changes in the kidneys, cardiovascular disease). The second section served to collect data about the acceptance of the disease, including glycemia regulation parameters (measuring of morning sugar values, postprandial sugar values, and HbA1c), awareness of the severity of the disease, current treatment (ability to specify the correct treatment and frequency of its use), and satisfaction with the diabetic care services.

For this research, clinical inertia was considered: last treatment change was six or more months ago and HbA1c < 7% shown by two measurements and/or increase of HbA1c in comparison to the previous visit and/or no HbA1c measurement, but glycemia < 7.2 mmol/L shown by two measurements and/or at least one measurement of glycemia < 7.2 mmol/L, and/or the patient has no information about last treatment change with HbA1c values > 7% and/or no measurements of glycemia nor HbA1c.

### 2.4. Statistical Analysis

Depending on the type of variables and the normality of the distribution, results are presented as frequency (percent), median (range), and mean ± sd. Methods used for testing the statistical hypotheses were the *t*-test, Mann–Whitney test, chi-square test, and Fisher’s exact test. Logistic regression was used to analyze binary outcomes (clinical inertia) and potential predictors. Independent variables that were significant (*p* < 0.05) in univariate logistic regression models were used as the independent variables in the multivariate logistic regression model. Statistical hypotheses were analyzed at the level of significance of 0.05. Statistical data analysis was performed using IBM SPSS Statistics 22 (IBM Corporation, Armonk, NY, USA).

## 3. Results

Our research sample included 543 patients, with an approximately even gender distribution (50.3% vs. 49.7%, male vs. female. respectively). The median age in the study population was 67 years, and more than half of our sample was living with a partner (69.1%). Questions about the socio-economic status of the participating patients showed that 54.6% had completed high/secondary school and an additional 10.3% with a lower level of education (Elementary school). Most of the patients (63.5%) were retired, and 51.7% reported average monthly income. Patients’ overall health condition was assessed as average by treating physicians in 51.4% of the sample, with the median duration of diabetes being ten years. Moreover, 372 patients (68.6%) had comorbidities along with diabetes. The general characteristics of patients included in the study population are presented in Table 1.

Corresponding physicians belonged to general practitioners (73.9%), predominantly female (86.3% female vs. 13.7% male), with a median of 19.5 years of work experience, and a median of 50 years of age. The majority of physicians also reported good or very good monthly incomes (27.3% and 29.5%).

After analyzing collected data, clinical inertia was identified in 224 patients or 41.3% of the research sample.

### 3.1. Patients’ Profiles and Clinical Inertia—Health Conditions and Behavior

Duration of diabetes appeared to be highly significant for clinical inertia. The median duration of diabetes diagnosis was 12 years in patients under clinical inertia, compared to the median ten years in those who showed no clinical inertia (*p* < 0.001).

Based on physicians’ assessment, patients with poor overall health conditions were more frequently subject to clinical inertia in their treatments (19.1% with and 11.9% without inertia, respectively, (*p* = 0.007).

Further, clinical inertia was more frequent in treating patients with a median of 2 comorbidities, compared to a median of 1 comorbidity, where no clinical inertia was observed (*p* < 0.001). Clinical inertia was observed in the treatment of 76.8% of patients with comorbidities, compared to 62.9% of patients without comorbidity, with the difference being highly significant (*p* = 0.001).

Body mass index (BMI) was also shown to impact the emergence of clinical inertia, with a mean BMI of 28.04 in the group with clinical inertia, compared to a mean of 27.68 in the group without inertia (*p* = 0.024).

At the same time, gender (*p* = 0.370), age (*p* = 0.981), marital status (*p* = 0.957), working status (*p* = 0.390), monthly income (*p* = 0.877), and educational level (*p* = 0.067) did not play a role in the presence of clinical inertia in the treatment of our study population (Table 1).

### 3.2. Phenomenon of Clinical Inertia in Type 2 Diabetes Treatment

Data related to an overall health condition and patients’ behavior—physical activity, diet, smoking, and alcohol consumption—were analyzed in two groups: patients with and without clinical inertia in their treatments (Table 2). Patients in the group with clinical inertia assessed their overall health condition significantly worse than those without clinical inertia. Only those related to the diet had significant differences between the two groups regarding patients’ behavior. In the group with clinical inertia, patients applied physicians’ recommendations for diet adjustment less. Diet was an important factor in differentiating patients into two groups (*p* = 0.006). After exploring the diet structure, the results pointed out that patients without clinical inertia incorporated fruits (*p* = 0.001) and vegetables (*p* = 0.005) in their diet. Physical activity (duration or level) had no significant difference between patients exposed and not exposed to clinical inertia during the treatment. Moreover, smoking (*p* = 0.600) and alcohol consumption (*p* = 0.700) did not show any correlation with clinical inertia (Table 2).

Analyzing the current treatment, the results showed that treatment with physical activity and diet (*p* = 0.556), metformin (*p* = 0.369), dipeptidyl peptidase-4 inhibitor (DPP-4 inhibitors) (*p* = 0.694), and sodium-glucose cotransporter one inhibitors (SGLT-2 inhibitors) (*p* = 0.086), short-acting human insulin was without significant difference in both groups: with and without clinical inertia. Though few patients received the treatment with sulfonylurea derivates, there was a significant difference (*p* = 0.026) between the two groups. Moreover, a significant difference in the two groups was present if we observed utilization of fixed mixtures of human insulin (6.7% in the group with clinical inertia and 2.8% in the group without inertia, *p* = 0.030). An even more significant difference was present in patients treated with modern insulin analogues, including short-acting analogues (22% vs. and 6%, respectively, *p* < 0.001), intermediate-acting analogues (35% vs. 14.2%, respectively, *p* < 0.001) and mixtures of analogues (10.8% vs. 4.1% respectively, *p* = 0.002). At the same time, observing the type of treatment and its intensification, results showed a significant difference between groups (*p* < 0.001). The number of OADs (without OAD 15.3%, 1 OAD 47.5% vs. 2 OAD 33.9% vs. 3 OADs 3.3%) did not significantly increase inertia (*p* = 0.191). On the contrary, adding insulin and intensifying therapy by adding additional insulin has been shown to significantly increase the odds for clinical inertia (*p* < 0.001). The relationship between current treatment and clinical inertia is shown in Table 3.

Patients in the two groups, i.e., with and without inertia, were not different in the availability of morning glycemia results (*p* = 0.151), glycemic profile (*p* = 0.450), or full profile (*p* = 0.179). On the other side, the frequency of HbA1c results showed the difference between groups (*p* = 0.003)—the group with clinical inertia had less regular testing results. We have observed similar results regarding lipid values (*p* = 0.001).

Differences between the two groups in frequency of patient monitoring parameters and clinical inertia are presented in Table 4.

Based on the data collected, most patients (53%) had their treatment adjustment more than a year ago, with an additional 19.3% of patients with a change over the previous six months.

As an important aspect of insulin treatment, the frequency of hypoglycemia, was analyzed. Patients who reported hypoglycemia episodes to their physicians were more present in the group with clinical inertia (27.7%) than in the group without inertia (17.5%) (*p* = 0.005). Further analysis of the daytime and nighttime hypoglycemic episodes did not show significance (*p* = 0.418) among the two groups. Similarly, there was no difference between the two groups with episodes confirmed by laboratory results (*p* = 0.870) and the group with severe hypoglycemia requiring hospitalization (*p* = 0.406).

Reflecting on healthcare system-related descriptors, the average duration of the medical examination was not different between groups with and without clinical inertia (*p* = 0.524). Additionally, the existence of the established treatment goals (*p* = 0.365) and visit schedules (*p* = 0.854) between the physician and the patient was also shown to have no significant difference. The same observations were found for the existence and use of diabetes counseling centers (*p* = 0.597), patient registry (*p* = 0.926), and special medical records (*p* = 0.387). However, analysis of patients’ referral to secondary-healthcare-level institutions pointed to significant differences between the two groups (*p* = 0.019). In our sample, 31.7% of patients with inertia vs. 42.0% without inertia were referred to the next-level healthcare center. This was not observed for referrals to the specialist unit at primary care (*p* = 0.956), counseling centers (*p* = 0.685), or the Clinical Center of Serbia (*p* = 0.453) (Table 5).

Prescription habits, individual changes of treatment (*p* = 0.563), initiation of the new medication (*p* = 0.70), combining OADs (*p* = 0.784), correction of OAD dose only (*p* = 0.131), initiation of insulin treatment (*p* = 0.625), insulin titration (*p* = 0.237), and combining insulins (*p* = 0.613) did not show a significant difference between patients with and without clinical inertia. Moreover, there was no difference between the two groups regarding the possibility of performing regular HbA1c measurement at primary healthcare centers (*p* = 0.384), educational activities with patients in healthcare centers (*p* = 0.324), educational activities by preventive centers (*p* = 0.375), educational activities by diabetes counseling units (*p* = 0.422), or educational activities by physicians (*p* = 0.233). Type of educational activity in patients with and without inertia did not show significant differences: in groups with individual education—37.7% vs. 38.4%, group education—3.0% vs. 1.4%, and both types of education—59.1% vs. 60.1% (*p* = 0.538). Similar results were observed in the scope of different educational materials. There were no significant differences between the two groups in the presence of educational material for self-control (*p* = 0.513), diet (*p* = 0.396), exercise (*p* = 0.167), or treatment of type 2 diabetes (*p* = 0.169).

We have collected data on the type of education (continuous medical education (CME), short presentations—industry initiated, and duration of education (up to 1 h, more than an hour, and a few days). Our results show that clinical inertia is more frequent among physicians attending organized educations only in healthcare institutions of their employment with the duration of up to one hour (educational events in the health institutions of employment: 49.58% and 30.8%, respectively, *p* = 0.034; up to one hour of duration: 62.3% and 47.7%, respectively, *p* = 0.001).

### 3.3. Predictors of Clinical Inertia—Multivariate Logistic Model

In this multivariate logistic regression model, we have included predictors of clinical inertia that were statistically significant in the univariate logistic regression models, with a significance level of *p* < 0.05. There is no significant multicollinearity between other predictors included in the multivariate model.

The model consists of 15 predictors presented in Table 6, which were compared with 421 patients, from which 166 patients had an outcome of interest—treatment with clinical inertia. Model in the whole (including all predictors) was significant (*p* < 0.001).

In this multivariate logistic regression model, several investigated factors were shown as significant predictors of clinical inertia, predominantly related to patients’ monitoring and therapeutical options.

Our analysis showed that patients who agree and comply with physician recommendations for dietary changes have 50% less chance of being clinically inert.

The frequency of providing HbA1c results, with an odds relationship of 0.3 (OR = 0.3), has shown that patients who regularly provide HbA1c results have 70% fewer odds for clinical inertia occurrence than those who never provide such results.

Patients on current treatment with short-acting insulin analogues have 4.5 times higher odds for experiencing clinical inertia. Treatment with intermediate-acting insulin analogues brings 2.6 times higher odds for clinical inertia (OR = 2.6), while patients on biphasic insulin analogues are 4.4 times more likely to experience clinical inertia (OR = 4.4).

Our results also demonstrated that, with each additional postponement of treatment change, chances for clinical inertia are increasing by 40%.

Patients who had their medication changed had 90% more chances of experiencing clinical inertia than those who had only their dose changed.

Patients referred to secondary healthcare institutions for further treatment adjustments had a 40% less chance for clinical inertia.

## 4. Discussion

First defined as an absence of a new drug administration or an absence of the change in the dose of an existing drug when necessary [10], clinical inertia has shown to be a complex phenomenon, influenced by many factors, and contributing to the development of the vast number of complications in patients suffering from chronic diseases, such as hypertension, dyslipidemia, diabetes, depression, and many more [11,13,15]. In the past, when the only options for diabetes treatment were metformin, sulfonylurea derivates, insulins, and their combinations, the fear of hypoglycemia was one of the main reasons for resistance in both physicians and patients [19,20]. Now, when more therapeutical choices are available, including glucagon-like peptide-1 receptor agonists (GLP-1 receptor agonists) and SGLT-2 inhibitors, the possibility to individualize treatment for each patient as per individual status has contributed to improvement in disease management and quality of life for patients with diabetes. The above-mentioned medication provides tighter glycemic control with less frequent hypoglycemic episodes [21,22]. At the same time, it allows us a more holistic approach, leading to less frequent hypoglycemic episodes, more comfort for patients, a positive effect on body weight, cardiovascular safety, and hopefully protectivity [23,24,25].

### 4.1. Challenges of Clinical Inertia in the Treatment of Type 2 Diabetes

Research published by Aujoulat et al., which explored factors associated with clinical inertia, reported that the presence of several concomitant pathologies, and therefore the use of multiple medications, may result in delay or absence of the needed intensification of treatment [26]. This work pointed out that physicians’ decisions depend on patients’ characteristics and medical history, such as patients’ age and health condition [26]. If we look only into the diabetes population, as per a paper published by Ruiz-Negron et al., clinical inertia was observed in over a third of type 2 diabetes patients with the uncontrolled disease and was most frequent in the groups of patients over 65 years of age, patients using ≥2 antihyperglycemic medications, ones who had an HbA1c 8.0–9.0%, and ones who had coronary heart disease. All these factors led to worse HbA1c outcomes [27]. Data collected in our research further support these observations, as overall poor health conditions assessed by physicians led to an increase of inertia (19.1% in patients exposed to clinical inertia vs. 11.9% in the group without inertia, *p* = 0.007). Regarding the patient’s overall health condition, its correlation with clinical inertia, presence, and the number of comorbidities was shown to play a significant role in physicians’ hesitation towards treatment change. Our data, as expected, show that the presence of comorbidities was higher in the group which had clinical inertia compared to patients without inertia (76.8% vs. 62.9%, respectively, *p* < 0.001). These observations can be explained by the fact that polypharmacy is present in patients with multiple diseases and conditions, which requires more attention and follow-up by physicians due to possible contraindications and drug–drug interactions. This situation may produce physicians’ hesitation to make changes, initiate new drugs, or decide to deviate from recommended titration algorithms (for example, prescribing a lower dose of insulin than recommended based on the glycemic values). All the above-mentioned factors require more physician’s time dedicated to each patient, which physicians do not have in most cases. Research exploring GPs’ workload in England showed that increased patient needs and expectations, along with changes in the healthcare system and downgrading some medical services from secondary to primary level, led to an overload of general practitioners [28]. This can be offered as one of the possible root causes of the already identified lack of time, which can significantly impact clinical inertia emergence. This challenge can be addressed as an issue and room for progress within the work organization and healthcare quality.

### 4.2. Patients’ Health Condition, Behavior and Clinical Inertia

Further into the analysis of patients’ condition, we have demonstrated that a longer duration of diabetes was present in the group exposed to clinical inertia (median 12 in the group with vs. 10 years, respectively, *p* < 0.001). Dietary habits (to which extent the patient is following dietary recommendation *p* < 0.006, intake of fruits *p* < 0.001, and vegetables *p* < 0.005) are worse in the group with observed clinical inertia in the treatment. This result is in line with another observation from our study, namely that treatment with clinical inertia is identified in patients with higher BMI. Since obesity is in tight correlation and frequently the following comorbidity in type 2 diabetes patients [29], our patients with higher average BMIs were also identified in the group exposed to clinical inertia (BMI 28.04 vs. 27.68, with vs. without inertia, respectively, *p* = 0.024).

Patient-related factors are no less important than physician-related factors regarding their impact on clinical inertia occurrence. It has been estimated that patient-related barriers account for ~30% of the main factors contributing to clinical inertia [11]. Throughout the literature, hypoglycemia is identified as one of the essential patient-related factors contributing to insulin avoidance and fear of insulin [30,31]. In a survey published by Polonsky et al., 43.3% of insulin-naive patients with type 2 diabetes stated that “problematic hypoglycemia” is one of their reasons for avoiding insulin [19]. Moreover, a survey including physicians worldwide found that if the risk of hypoglycemia was not a concern, most clinicians (75.5%) would be more relaxed in the intensification of insulin therapy [20]. Our research showed that clinical inertia in treatment is substantial in patients reporting hypoglycemia episodes to their physicians, compared with patients with fewer reported episodes who belonged to the group without clinical inertia (27.7% vs. 17.5%, respectively, *p* = 0.005). The time of the day when a hypoglycemic episode was experienced did not significantly impact clinical inertia. This finding is in accordance with what we can see in other papers, as hypoglycemia is one of the most frequent reasons for repulsion to initiate or intensify insulin treatment [19,20].

### 4.3. Current Treatment and the Clinical Inertia

One of the main focuses of our research was current treatment and its correlation with clinical inertia. The collected data did not show that treatment with physical activity and diet, metformin, DPP-4 inhibitors, SGLT-2 inhibitors, short-acting human insulins, and intermediate-acting human insulin were related to clinical inertia. Interestingly, patients treated with sulfonylurea derivates were present more in the group not exposed to clinical inertia (*p* = 0.026). Given that sulfonylurea derivates are still a widely used treatment in Serbia, primarily due to low price, this topic could represent an area for further research.

We have identified that the current utilization of fixed mixtures of human insulin is higher in the patient group exposed to treatment with clinical inertia (6.7% vs. 2.8%, respectively, *p* = 0.030) and even more significant in patients treated with modern insulin analogues. There are several possible explanations for this finding. One explanation is that currently, in Serbia, modern insulins are the last instance in the treatment of patients with type 2 diabetes, and intensified insulin treatment is considered for all patients not achieving good glycemic control with other treatment options [7]. This may lead treating physicians to question “What is the next step?” after insulin intensification in these patients. In a paper published by Ling-Wang et al., factors related to inertia, besides insulin treatment, include treatment change, poor adherence to diet, exercise, self-measured blood glucose (SMBG) during follow up period, and <3 HbA1c measurements per year [32]. The results of our research are partially in line with this study, as the frequency of HbA1c measurements showed a significant difference between groups (*p* = 0.003). Alternatively, the frequency of morning glycemia monitoring, glycemic profile, or full profile did not pose a significant difference between patients exposed to treatment with and without clinical inertia. As per the National Guideline for Diabetes Care, glycemia values, HbA1c, and lipids should be examined on each regular visit, which is recommended at least once every 3–4 months [7]. Considering that simple glycemia values are part of routine laboratory practice, and HbA1c and lipid profiles are more specific analyses, this may pose a question if physicians are requesting patients to examine these parameters to a sufficient extent and consider them for making treatment decisions. Additionally, this can question the health system itself and whether the system of diabetic care is making these analyses available for all patients in need.

Inertia significantly rose as the number of antidiabetic drugs was higher. When the data were stratified to OAD and insulin, it was observed that an increase in clinical inertia could be attributed to the addition of insulin rather than OAD. No difference in clinical inertia was observed between groups using 1, 2, or 3 OADs, unlike in the group using insulin, where the odds for clinical inertia increased significantly with initiating insulin or the addition of another insulin (insulin intensification). Several works reflect this topic. Work by Ruiz-Negrón et al. demonstrated that having two, three, or more antihyperglycemic medications was associated with higher chances of inertia [27]. This correlation between clinical inertia and insulin therapy can be, at least partially, explained by the fear of hypoglycemia, weight gain, injections, influence on patients’ everyday life, and treatment complexity, which was pointed out in other papers researching this phenomenon [33,34].

Our research demonstrates that most patients (29.7%) had treatment adjustment more than a year ago, 15.1% two years ago, and 17.9% three years ago, with an additional 19.2% of patients with a change over the previous six months. Studying the nature of the change itself, most of the analyzed patients had only received dose adjustments (43.1%) but remained on the same medication. In 31.1% of patients, the physician decided to initiate additional medication, and, in 25.9% of observed patients, medication was changed. Therefore, clinical inertia is significant when deciding to initiate additional or completely substitute the current treatment. This delay is in line with results of previously published articles, showing that a delay in the change of treatment, depending on the healthcare system, disease progression, and background medication can, be several years [11,14].

### 4.4. Healthcare System Descriptors and Clinical Inertia

It is necessary to reflect on healthcare system descriptors as another significant factor influencing clinical inertia. Several interesting findings were derived from our research. The average duration of the medical examination was shown to have no impact on clinical inertia occurrence. Maybe even more interesting was the fact that diabetes counseling centers, patient registry, special medical records, and medication change also showed no significant impact on inertia in type 2 diabetes patients. Since these assets were developed and provided as supporting and educational tools for diabetes patients, their non-significance in reducing clinical inertia may lead to the question of whether they are being used in their total capacity. The existence of educational support to patients should make a significant difference in disease management, and lack of proof may indicate that clinical inertia is present in physicians and treatment of their patients and in the healthcare system itself. Alternatively, patient referral to higher-level healthcare institutions significantly reduced clinical inertia occurrence. Our study demonstrated a significant difference (*p* = 0.019)—68.3% of the patients not referred to the secondary healthcare level institutions belonged to the group with clinical inertia in the treatment, compared to 58% of the patients in another group. Patient referral can also be questioned in the scope of the healthcare system, as well as accessibility to the more specialized institutions and physicians, especially taking into consideration limitations for insulin initiation at the primary healthcare level. Based on the results of our research, the broader possibilities for diabetes care, available at higher level healthcare institutions, contribute to this finding. In the work by Schernthaner et al., it was proposed that physicians on the primary-care level have fewer education opportunities on diabetes and other chronic diseases. Therefore, clinical inertia is identified more frequently [35]. Although our work did not reflect on medical professionals other than physicians, studies that explored the coordination between different medical staff have demonstrated that this type of cooperation can also impact clinical inertia occurrence [36]. Hirsch et al. reported that patients with type 2 diabetes mellitus and complex health conditions and comorbidities who were treated by collaborating pharmacist and endocrinologist achieved HbA1c reduction of 2.4% within 6 months [37].

The dilemma for treating physicians and questioning where to go next if intensified insulin treatment is still not enough to bring patients into good control can potentially be related to education and educational activities provided to physicians [10,11]. Interestingly, our results show that physicians attending education in the form of presentations (*p* = 0.001), which includes education lasting up to 1 h (*p* = 0.034), mostly promotion-focused, and at the same time the most common form of education, are more likely to be clinically inert. Based on our research, education for several hours to 1 day is shown to be the most effective. There are more possible explanations for this. In our opinion, education lasting for one hour is either performed before the shift, immediately after the shift, or in the break, which questions physicians’ motivation and willingness to participate. The second dimension is the content of this education, mainly related to drugs with limited impact on the overall disease-treatment approach. Taking into account that physician education is one of the cornerstones mentioned in several papers [10,11], this should be one of the important focus areas in the battle to defeat clinical inertia.

### 4.5. Strengths and Limitations of the Study

The strengths of our study include the fact that data were collected in an environment that patients to frequently visit. It was designed to collect data from “typical” patients and treating physicians during regular visits and explore real-life surroundings and routine clinical practice. Within this approach, we consider results to represent the current status and position of the healthcare system and medical care provided to patients suffering from type 2 diabetes mellitus. Additionally, the sample size of over 500 patients, covering several variables, provides a comprehensive insight into the population.

There are several limitations to our study. We were not questioning whether treatment was adjusted during the current visit, nor how patients are positioned in comparison to defined goals, which would be interesting to explore as an important describer of clinical inertia. Since our research was conducted during the COVID-19 pandemic, patients were referred from institutions included in the COVID-19 system, leading to an increased number of patients and therefore reduced time for the individual exam. Additionally, the length of the questionnaire could be considered as a limitation, due to time constraints. It is worth mentioning that we were not taking into consideration treatment reduction or termination as a type of clinical inertia. This appearance could be of significant importance to be addressed when new therapeutical options become available. Moreover, our research could not follow up with potential alternative communication channels between healthcare providers and patients, such as “over the phone consultancy,” which was an important communication channel during COVID-19 lockdowns. Nevertheless, the study has still achieved its imposed aim to assess the level of clinical inertia during type 2 diabetes treatment and provide recommendations for overcoming some of the barriers connected to clinical inertia.

## 5. Conclusions

The phenomenon of clinical inertia is relatively new in scientific circles. So far, the literature has been dominated by discussions that refer to the identification and definition of the concept and determination of the level of consequences that clinical inertia brings. There are attempts, although very few, to assess inertia and reduce its effects.

Our findings in Serbia are in line with results reported over the years from different countries. We have shown that various factors related to the healthcare system, physicians, and patients, such as overall health condition, comorbidities, diabetes duration, current treatment, last treatment change, HbA1c and FPG measuring frequency, BMI, patient referral, diet adjustment, and physician education, play a role in the development of clinical inertia.

There are several possible solutions that can contribute to overcoming this widespread phenomenon. One of them could be educating the physicians, providing them with comprehensive and up-to-date information and knowledge, to use maximum resources and treatment options available to answer patient needs, especially those on insulin treatments. No less important is educating patients, where general education is the foundation for more specific knowledge and skills.

## Figures and Tables

**Table 1:** General characteristics of participating patients and related clinical inertia.

Variable	Total (n = 543)	Clinical Inertia (n = 224)	Without Clinical Inertia (n = 319)	*p*-Value
Gender, n (%)				0.370
Female Male	268 (49.7%) 271 (50.3%)	116 (52.0%) 107 (48.0%)	152 (48.1%) 164 (51.9%)	
Age, Median (range)	67 (31–86)	67 (31–85)	67 (31–86)	0.981
BMI, Mean (range)	28.25 (16.4–45.0)	28.77 (18.5–45.0)	27.89 (16.4–45)	0.024
Marital status, n (%)				0.957
Married/living with partner Living alone Divorced Widower/widow	354 (69.1) 50 (9.8%) 31 (31%) 77 (15%)	150 (68.8%) 22 (10.1%) 12 (5.5%) 34 (15.6%)	204 (69.4%) 28 (9.5%) 19 (6.5%) 43 (14.6%)	
Working status, n (%)				0.390
Temporary work Permanent work Unemployed Retired	130 (25.3%) 23 (4.5%) 34 (6.6%) 326 (63.5%)	52 (23.7%) 12 (5.5%) 11 (5.0%) 144 (65.8%)	78 (26.5%) 11 (3.7%) 23 (7.8%) 182 (61.9%)	
Educational level, n (%)				0.067
Elementary school High/Secondary school College Faculty	52 (10.3%) 277 (54.6%) 86 (17.0%) 92 (18.1%)	23 (10.6%) 128 (59.0%) 35 (16.1%) 31 (14.3%)	29 (10.0%) 149 (51.4%) 51 (17.6%) 61 (21%)	
Patients’ estimation of monthly income, n (%)				0.877
Very low Low Average Good Very good	16 (3.1%) 72 (14.1%) 263 (51.7%) 125 (24.6%) 33 (6.5%)	7 (3.3%) 26 (12.1%) 118 (54.9%) 55 (25.6%) 9 (4.2%)	9 (3.1%) 46 (15.6%) 145 (49.3%) 70 (23.8%) 24 (8.2%)	
Overall health condition (physicians’ estimation), n (%)				0.007
Very poor Poor Average Good Very good	3 (0.6%) 80 (14.8%) 277 (51.4%) 169 (31.4%) 10 (1.9%)	2 (0.9%) 42 (19.1%) 114 (51.8%) 56 (26.8%) 3 (1.4%)	1 (0.3%) 38 (11.9%) 163 (51.1%) 110 (34.5%) 7 (2.2%)	
Diabetes duration, Median (Range)	10 (1–46)	12 (1–46)	10 (1–40)	<0.001
Presence of comorbidities, n (%)				0.001
No Yes	170 (31.4%) 372 (68.6%)	52 (23.2%) 172 (76.8%)	118 (37.1%) 200 (62.9%)	

BMI, body mass index.

**Table 2:** Overall health condition and patients’ behavior in relation to clinical inertia in the treatments of type 2 diabetes.

Variables	Total (n = 543)	Clinical Inertia (n = 224)	Without Clinical Inertia (n = 319)	*p*-Value
Overall health condition (patients’ estimation), n (%)				<0.001
Very poor Poor Average Good Very good	4 (0.8%) 68 (12.8%) 266 (50.1%) 169 (31.8%) 24 (4.5%)	4 (1.8%) 39 (17.8%) 112 (51.1%) 57 (26.0%) 7 (3.2%)	0 (0.0%) 29 (9.3%) 154 (49.4%) 112 (35.9%) 17 (5.4%)	
Type of physical activity, n (%)				0.101
Without Mild Moderate Intense	44 (8.2%) 388 (72.3%) 53 (9.9%) 52 (9.7%)	19 (8.6%) 167 (75.6%) 19 (8.6%) 16 (7.2%)	25 (7.9%) 221 (69.9%) 34 (10.8%) 36 (11.4%)	
Diet adjustments according to physician suggestions, n (%)				0.006
I am not following it As possible Fully	40 (7.6%) 389 (73.7%) 99 (18.8%)	18 (8.4%) 170 (79.4%) 26 (12.1%)	22 (7.0%) 219 (69.7%) 73 (23.2%)	
Fruit consumption, n (%)				0.001
Never Less than once monthly 1–3 times per week 4–6 times per week Once daily Two or more times per day	4 (0.7%) 32 (6.0%) 108 (20.2%) 98 (18.3%) 214 (40.0%) 79 (14.8%)	1 (0.5%) 15 (6.8%) 59 (26.8%) 43 (19.5%) 78 (35.5%) 24 (10.9%)	3 (1.0%) 17 (5.4%) 49 (15.6%) 55 (17.5%) 136 (43.2%) 55 (17.5%)	
Vegetable consumption, n (%)				0.005
Never Less than once monthly 1–3 times per week 4–6 times per week Once daily Two or more times per day	1 (0.2%) 16 (3.0%) 72 (13.5%) 93 (17.4%) 204 (38.1%) 149 (27.9%)	1 (0.5%) 8 (3.6%) 35 (15.9%) 41 (18.6%) 88 (40.0%) 47 (21.4%)	0 (0.0%) 8 (2.5%) 37 (11.7%) 52 (16.5%) 116 (36.8%) 102 (32.4%)	
Smoking status, n (%)				0.600
Yes, regularly Yes, from time to time No	82 (15.5%) 43 (8.1%) 403 (76.3%)	30 (13.7%) 19 (8.7%) 170 (77.6%)	52 (16.8%) 24 (7.8%) 233 (75.4%)	
More than 6 alcoholic drinks in last 12 months, n (%)				0.700
Never Once per month Once per week Every day or almost every day	401 (76.4%) 87 (16.6%) 33 (6.3%) 4 (0.8%)	166 (75.6%) 35 (16.0%) 15 (6.8%) 3 (1.4%)	235 (76.8%) 52 (17.0%) 18 (5.9%) 1 (0.3%)	

**Table 3:** Diabetes type 2 treatment characteristics and related clinical inertia.

Variables	Total (n = 543)	Clinical Inertia (n = 224)	Without Clinical Inertia (n = 319)	*p*-Value
Current treatment: Exercise and diet, n (%) No Yes				0.556
	218 (40.2%) 324 (59.8%)	93 (41.7%) 130 (58.3%)	125 (39.2%) 194 (60.8%)	
Current treatment: Metformin, n (%) No Yes				0.369
	95 (17.5%) 447 (82.5%)	43 (19.3%) 180 (80.7%)	52 (16.3%) 267 (83.7%)	
Current treatment: Sulfonylureas, n (%) No Yes				0.026
	357 (65.9%) 185 (34.1%)	159 (71.3%) 64 (28.7%)	198 (62.1%) 121 (37.9%)	
Current treatment: DPP-4 inhibitors, n (%) No Yes				0.694
	536 (98.9%) 6 (1.1%)	220 (98.7%) 3 (1.3%)	316 (99.1%) 3 (0.9%)	
Current treatment: SGLT-2 inhibitors, n (%) No Yes				0.086
	499 (92.1%) 43 (7.9%)	200 (89.7%) 23 (10.3%)	299 (93.7%) 20 (6.3%)	
Current treatment: GLP-1 RA, n (%) No Yes				0.411
	541 (99.8%) 1 (0.2%)	222 (99.6%) 1 (0.4%)	319 (100%) 0 (0.0%)	
Current treatment: Human insulin Short-acting, n (%) No Yes				0.283
	518 (95.7%) 23 (4.3%)	216 (96.9%) 7 (3.1%)	302 (95.0%) 16 (5.0%)	
Current treatment: Human insulin Basal, n (%) No Yes				0.709
	477 (88.2%) 64 (11.8%)	198 (88.8%) 25 (11.2%)	279 (87.7%) 39 (12.3%)	
Current treatment: Human insulin Biphasic, n (%) No Yes				0.030
	517 (95.6%) 24 (4.4%)	208 (93.3%) 15 (6.7%)	309 (97.2%) 9 (2.8%)	
Current treatment: Insulin analogue Short-acting, n (%) No Yes				<0.001
	473 (87.4%) 68 (12.6%)	174 (78.0%) 49 (22.0%)	299 (94.0%) 19 (6.0%)	
Current treatment: Insulin analogue Basal, n (%) No Yes				<0.001
	418 (77.3%) 123 (22.7%)	145 (65.0%) 78 (35.0%)	273 (85.8%) 45 (14.2%)	
Current treatment: Insulin analogue Biphasic, n (%) No Yes				0.002
	504 (93.2%) 37 (6.8%)	199 (89.2%) 24 (10.8%)	305 (95.9%) 13 (4.1%)	
Type of current treatment, n (%)				<0.001
Exersise and diet OAD OAD combinations (2–3) Basal insulin +/− OAD(s) Intensified insulin treatment +/− OAD(s)	11 (2%) 132 (24.3%) 159 (29.3%) 139 (25.6%) 102 (18.8%)	5 (2.2%) 29 (12.9%) 53 (23.7%) 77 (34.4%) 60 (26.8%)	6 (1.9%) 103 (32.3%) 106 (33.2%) 62 (19.4%) 42 (13.2%)	

DPP-4 inhibitors, Dipeptidyl peptidase-4 inhibitor; SGLT-2 inhibitors, Sodium-glucose cotransporter 1 inhibitors; GLP-1 RA, Glucagon-like peptide-1 receptor agonists; OAD, oral antidiabetic drug.

**Table 4:** Frequency of patients’ monitoring parameters and related clinical inertia.

Variables	Total (n = 543)	Clinical Inertia (n = 224)	Without Clinical Inertia (n = 319)	*p*-Value
Laboratory FPG result frequency, n (%)				0.151
Never From time to time Regularly	54 (10.2%) 326 (61.6%) 149 (28.2%)	20 (9.3%) 145 (67.4%) 50 (23.3%)	34 (10.8%) 181 (57.6%) 99 (31.5%)	
FPG at current visit (mmol/L), Mean (Range)	7.92 (2.6–27.6)	8.65 (3.80–27.60)	7.43 (2.6–17.00)	
Laboratory HbA1c result frequency, n (%)				0.003
Never From time to time Regularly	29 (5.9%) 354 (72.2%) 107 (21.8%)	15 (7.7%) 151 (77.0%) 30 (15.3%)	14 (4.8%) 203 (69.0%) 77 (26.2%)	
HbA1c at current visit (%), Mean (Range)	7.32 (5.03–14)	8.02 (7.07–17.00)	6.83 (5.03–11.35)	
Providing glycemia profile at the visit, n (%)				0.450
Never From time to time Regularly	170 (31.7%) 314 (58.5%) 53 (9.9%)	61 (27.9%) 141 (64.4%) 17 (7.8%)	109 (34.3%) 173 (54.4%) 36 (9.9%)	
Is glycemia profile complete, n (%)				0.179
No Sometimes Yes	168 (35.1%) 108 (22.5%) 203 (42.4%)	79 (38.7%) 44 (21.6%) 81 (39.7%)	89 (32.4%) 64 (23.3%) 122 (44.4%)	
Providing lipid values at the visit, n (%)				0.001
Never From time to time Regularly	15 (2.8%) 404 (75.4%) 117 (21.8)	8 (3.7%) 178 (81.7%) 32 (14.7%)	7 (2.2%) 226 (71.1%) 85 (26.7%)	
Patient is reporting hypoglycemia, n (%)				0.005
No Yes	413 (78.2%) 115 (21.8%)	159 (72.3%) 61 (27.7%)	254 (82.5%) 54 (17.5%)	

FPG, fasting plasma glucose; HbA1c, glycosylated hemoglobin.

**Table 5:** Healthcare system related descriptors and clinical inertia.

Variables	Total (n = 543)	Clinical Inertia (n = 224)	Without Clinical Inertia (n = 319)	*p*-Value
Average duration of examination, Median (Range)	15 (5–45)	15 (5–45)	15 (5–40)	0.524
Agreed goal of treatment, n (%)				0.365
No Yes	24 (4.6%) 499 (95.4%)	12 (5.6%) 203 (94.4%)	12 (3.9%) 296 (96.1%)	
Predefined visit plan, n (%)				0.854
No Yes	92 (17.7%) 429 (82.3%)	37 (17.3%) 177 (82.7%)	55 (17.9%) 252 (82.1%)	
Presence of counseling unit, n (%)				0.597
No Yes	294 (56.0%) 231 (44.0%)	118 (54.6%) 98 (45.4%)	176 (57.0%) 133 (43.0%)	
Existence patient registry, n (%)				0.926
No Yes	54 (10.5%) 459 (89.5%)	22 (10.4%) 190 (89.6%)	32 (10.6%) 269 (89.4%)	
Special Medical record (counseling unit/preventive center), n (%)				0.387
No Yes	180 (35.3%) 330 (64.7%)	78 (37.5%) 130 (62.5%)	102 (33.8%) 200 (66.2%)	
For further treatment adjustment patients are referred to specialist unit at primary health center, n (%)				0.956
No Yes	346 (67.4%) 167 (32.6%)	140 (67.3%) 68 (32.7%)	206 (67.5%) 99 (32.5%)	
For further treatment adjustment patients are referred to Clinical Hospital Center, n (%)				0.019
No Yes	319 (62.2%) 194 (37.8%)	142 (68.3%) 66 (31.7%)	177 (58.0%) 128 (42.0%)	
For further treatment adjustment patients are referred to University Clinical Center of Serbia, n (%)				0.453
No Yes	328 (63.9%) 185 (36.1%)	137 (65.9%) 71 (34.1%)	191 (62.6%) 114 (37.4%)	
For further treatment adjustment patients are referred to counseling units, n (%)				0.685
No Yes	345 (67.3%) 168 (32.7%)	142 (68.3%) 66 (31.7%)	203 (66.6%) 102 (33.4%)	

**Table 6:** Predictors of clinical inertia in of type 2 diabetes treatment.

Variables	B	*p*	OR	95% CI Lower	95% CI Upper
BMI	0.037	0.189	1.04	0.98	1.10
Diet adjustments according to physician suggestions	−0.734	0.003	0.5	0.3	0.8
Patient overall health condition, physicians’ estimation	−0.127	0.439	0.9	0.6	1.2
Number of comorbidities	0.020	0.843	1.0	0.8	1.2
Diabetes duration	−0.006	0.767	0.99	0.96	1.03
Frequency of FPG measurement	0.080	0.300	1.1	0.9	1.3
Laboratory HbA1c result frequency (never)	ref.				
Laboratory HbA1c result frequency (occasionally)	−0.153	0.758	0.9	0.3	2.3
Laboratory HbA1c result frequency (regularly)	−1.164	0.047	0.3	0.1	1.0
Current treatment-Human insulin fixed mix	1.020	0.085	2.8	0.9	8.9
Current treatment-Insulin analogue short-acting	1.499	0.002	4.5	1.8	11.3
Current treatment-Insulin analogue basal	0.960	0.011	2.6	1.2	5.5
Current treatment-Insulin analogue biphasic	1.491	0.003	4.4	1.6	12.0
Last treatment change	0.310	<0.001	1.4	1.2	1.6
Last treatment change (dose change of the same medication)	ref.				
Last treatment change (medication change)	−0.002	0.995	1.0	0.6	1.8
Last treatment change (adding additional drug on current treatment)	0.643	0.019	1.9	1.1	3.3
Patient referral to Clinical Hospital Center	−0.560	0.023	0.6	0.4	0.9
Physician’s continuing education about diabetes	−0.037	0.907	1.0	0.5	1.8

BMI, body mass index; FPG, fasting plasma glucose; HbA1c, glycosylated hemoglobin.

## Data Availability

The data presented in this study are available upon request from the corresponding author.
